# ﻿On *Psalmopoeus* Pocock, 1895 (Araneae, Theraphosidae) species and tarantula conservation in Ecuador

**DOI:** 10.3897/zookeys.1186.108991

**Published:** 2023-12-13

**Authors:** Pedro Peñaherrera-R., Roberto J. León-E.

**Affiliations:** 1 Universidad San Francisco de Quito USFQ, Colegio de Ciencias Biológicas y Ambientales, Instituto de Biodiversidad Tropical IBIOTROP, Laboratorio de Zoología Terrestre, Museo de Zoología, Quito 170901, Ecuador Universidad San Francisco de Quito Quito Ecuador

**Keywords:** Andes, Choco, pet trade, Psalmopoeinae, smuggling, taxonomy, western Ecuador

## Abstract

Two novel species of *Psalmopoeus* Pocock, 1895 are described from the north-western and central-western slopes of the Cordillera Occidental of the Andes mountain range in Ecuador. The new species are easily differentiated from other congeners of *Psalmopoeus* by spermathecae and male palpal bulb morphology and a comparatively distant distribution to the type localities of the geographically nearest known congeners. The diagnosis of *P.ecclesiasticus* Pocock, 1093 is revised and updated, considering the novel species and observations on spermatheca of this species. Likewise, an evaluation is provided for the new species in terms of conservation due to the various threats impacting ecosystems and ecosystem services of their type localities. Finally, the importance of theraphosid spiders in Ecuador and South America and their possible conservation requirements are discussed and assessed.

## ﻿Introduction

*Psalmopoeus* Pocock, 1895 includes arboreal Psalmopoeinae spiders Samm & Schmidt, 2010 diagnosed from all other members of the subfamily Psalmopoeinae mainly by the presence of maxillary lyra with one row of thick and rough stridulatory setae (Cifuentes and Bertani 2022). This arboreal clade currently has ten valid species distributed largely in Central America down to South America; Ecuador is the southernmost record for the genus with *Psalmopoeusecclesiasticus* Pocock, 1903, with the Antilles (Cifuentes and Bertani 2022; WSC 2023).

Literature on Ecuadorian Psalmopoeinae spiders is scarce, with only three valid species described from the country (WSC 2023). These are distributed along the western slopes of Cordillera Occidental of the Andes, the eastern slopes of the Cordillera Real Oriental of the Andes, and lowland Amazonia, *Psalmopoeusecclesiasticus* Pocock, 1903, *Amazoniuselenae* (Schmidt, 1994), and *Tapinaucheniuscupreus* Schmidt & Bauer, 1996 respectively (Pocock 1903; Hüsser 2018; Gabriel and Sherwood 2019; Cifuentes and Bertani 2022). This paper aims to describe two new species of *Psalmopoeus* recently discovered in the Province of Cotopaxi in the central-western region of Ecuador, the Province of Santo Domingo de Los Tsachilas, and the Province of Pichincha in western Ecuador. Additionally, we provide the first commentaries and suggestions about the conservation of tarantulas in Ecuador and the possible threats that Theraphosidae may face in terms of extinction.

## ﻿Materials and methods

Examined specimens are deposited at Museo de Zoología, Universidad San Francisco de Quito, Ecuador (**ZSFQ-i**) and Museo de Zoología, Pontificia Universidad Católica del Ecuador (**QCAZ-I**). Information on species for comparative diagnoses were obtained from actual redescriptions and descriptions of *Psalmopoeus* species (Mendoza 2014; Gabriel and Sherwood 2019; Cifuentes and Bertani 2022).

Specimens from ZSFQ-i were examined and measured under an Olympus SZX16 stereomicroscope with an Olympus DP73 digital camera. Specimens from QCAZ-I were examined and measured under a Nikon SMZ745T stereomicroscope with an Mshot MS60. All measurements are presented in millimetres. Female genitalia were excised using a syringe tip; soft tissue was digested with a solution of 15% KOH, washed in distilled water and 75% ethanol, and examined under an Olympus SZX16 stereomicroscope. Compound images were obtained by stacking a series of photographs taken at different depths processed with the staking software of Photoshop and editing tools.

Biogeographic classification follows the proposal by Morrone (2014), with modifications proposed by Cisneros-Heredia and Yánez-Muñoz (2007) and Cisneros-Heredia (2006, 2007, 2019). Ecuadorian classification of ecosystems follows MAE (2013). Shape files of cropland use and mining concessions were extracted from Potapov et al. (2022) and Peñaherrera-Romero (2023). Conservation categories and criteria follow IUCN (2001).

General description and measurements follow standards proposed by Gabriel and Sherwood (2019) and Cifuentes and Bertani (2022) for the genus *Psalmopoeus*. Bulb length proportion follows measurements proposed by Cifuentes and Bertani (2022). Leg spination description follows Cifuentes and Bertani (2022). The term longitudinal folds follows, in part, Dupérré and Tapia (2020).

The type locality and historical distribution of *P.ecclesiasticus* was obtained from the original description of Pocock (1903) with additional information from Peters (1955), Lynch and Duellman (1997), and Gabriel and Sherwood (2019). Additional records of *P.ecclesiasticus* distribution were obtained from the examined specimen deposited in the QCAZ-I collection and Cifuentes and Bertani (2022) records.

Morphological somatic abbreviations: **AME**, anterior median eyes; **ALE**, anterior lateral eyes; **PME**, posterior median eyes; **PLE**, posterior lateral eyes. Female: **iLB**, ill-defined lobe; **wLB**, well-defined lobe.

## ﻿Taxonomic account

**Theraphosidae Thorell, 1869**

**Psalmopoeinae Samm & Schmidt, 2010**

***Psalmopoeus* Pocock, 1895**

### ﻿ Psalmopoeus chronoarachne sp. nov.

Taxon classificationAnimaliaAraneaeTheraphosidae

EE09F452-E3F0-5CC9-AAC4-47E8D65B91A3

https://zoobank.org/F87ABBB2-B8AB-44F0-A788-BA0DFFC51342

Figs 1 , 2 , 3

#### Material examined.

***Holotype***: Republic of Ecuador • 1 ♀; Province of Cotopaxi, Canton Pangua, Parish of El Corazón, Hacienda La Mariela; -1.0856, -79.1841, 760 m a.s.l.; 27 February 2023; M. López-García, J. Montalvo, D. Brito-Zapata and C. Reyes-Puig leg.; ZFSQ-i11704.

#### Diagnosis.

**Figure 1.**
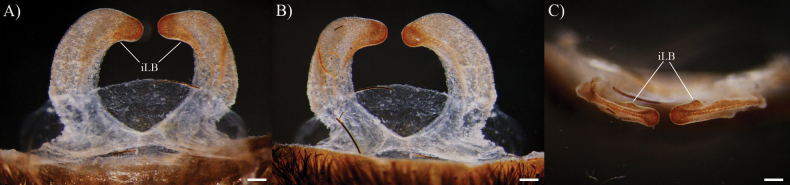
*Psalmopoeuschronoarachne* sp. nov. female holotype (ZSFQ-i11704), spermatheca **A** dorsal view **B** ventral view **C** apical view. Abbreviation: iLB, ill-defined lobe. Scale bars: 0.2 mm.

*Psalmopoeuschronoarachne* sp. nov. can be distinguished from its known congeners by spermathecal morphology, specifically: from *Psalmopoeussatanas* sp. nov. by having only a single ill-defined lobe on each receptacle, absence of well-defined lobes, apical digitiform lobe, comparatively receptacles more curved towards the centre (Fig. 1) (two ill-defined lobe and a single domed well-defined lobe in receptacles, apical digitiform lobe present and receptacles comparatively less curved towards the centre in *P.satanas* sp. nov.; Fig. 8); from *P.ecclesiasticus* by having comparatively less curved receptacles towards the centre and distant to each other, distal apex more curved and not overlapping, receptacles with only a single ill-defined lateral lobe on apical-inner and apical digitiform lobe absent (Fig. 1) (comparatively more curved receptacles towards the centre, distal apex less curved and overlapping, receptacles with apical digitiform lobe, one to four protruding well-defined lobes, and a single ill-defined lobe in *P.ecclesiasticus*; Fig. 10; see also Gabriel and Sherwood 2019: fig. 1; Cifuentes and Bertani 2022: figs 224, 229); from *P.cambridgei*, *P.irminia*, *P.pulcher*, *P.langenbucheri*, *P.reduncus*, and *P.victori* by having elongated and curved receptacles towards the centre with distal apex curved with only a single ill-defined lateral lobe on apical-inner and apical digitiform lobe absent (elongated and straight receptacles with distal apex straight with apical digitiform lobe and various central lobes in *P.cambridgei*, *P.irminia*, *P.pulcher*; elongated and triangular receptacles with distal apex straight, comparatively more sclerotised, thinner, and shorter apical digitiform lobe pointing upwards, not overlapping but very close and two to three well-defined lateral lobes in *P.langenbucheri*; short and triangular receptacles with distal apex straight, comparatively more elongated apical digitiform lobe pointing upwards but not overlapping and only a single ill-defined lateral lobe in *P.reduncus*; elongated and straight receptacles with distal apex slightly curved upwards or straight without receptacles in *P.victori*; see figures in Mendoza 2014: figs 27, 28; Cifuentes and Bertani 2022: figs 125, 170–175, 190–191, 215, 245, 268–271, 283, 300, 309).

#### Description.

**Figure 2.**
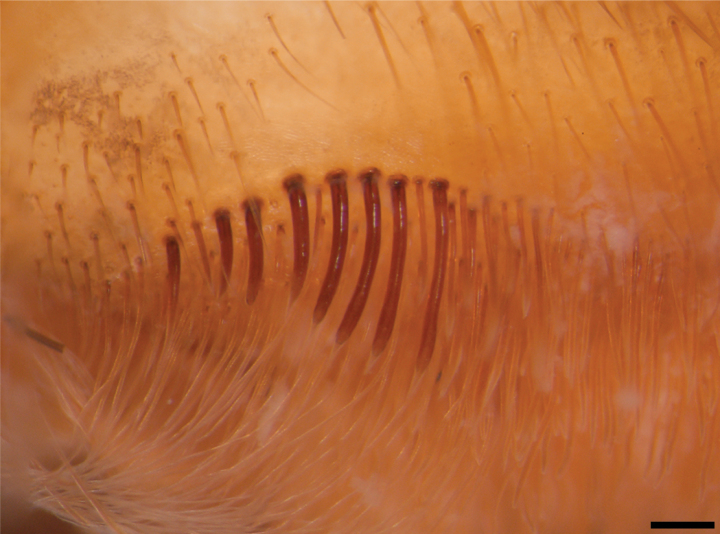
*Psalmopoeuschronoarachne* sp. nov. female holotype (ZSFQ-i11704): Maxillae (left side) showing maxillary lyra. Scale bar: 0.2 mm.

**Figure 3.**
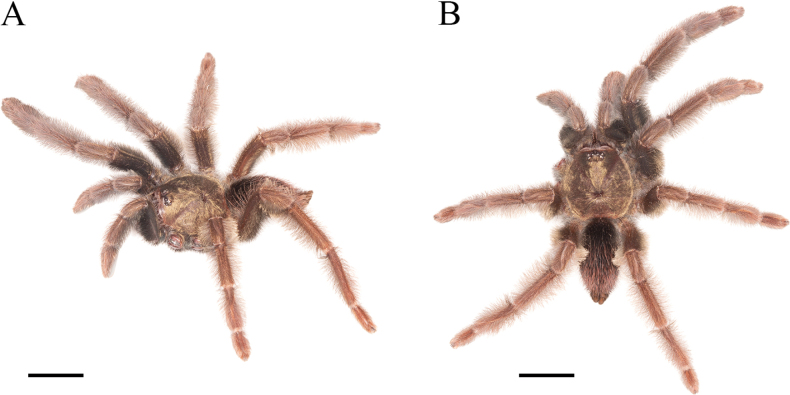
*Psalmopoeuschronoarachne* sp. nov. female holotype (ZSFQ-i11704), live habitus **A** dorsolateral view **B** dorsal view. Scale bars: 10 mm.

**Female holotype** (ZSFQ-i11704): Total length including chelicerae: 30.48. Carapace: length 11.84, width 10.50. Caput: slightly raised. Ocular tubercle: slightly raised, length 1.34, width 3.01. Eyes: ALE > AME, AME > PLE, PLE > PME, anterior eye row straight, posterior row slightly recurved. Clypeus: wide; clypeal fringe long. Fovea: straight. Chelicera: length 6.45, width 2.70. Abdomen: length 12.19, width 6.84. Maxilla with 147–224 cuspules covering approximately 30% of the proximal edge. Labium: length 2.05, width 1.65, with 163 cuspules most separated by 1.0–2.0× the width of a cuspule. Labio-sternal mounds joined along the entire base of the labium. Sternum: length 5.33, width 4.42, with two pairs of sigilla. Tarsi I–IV fully scopulate, tarsi I and II divided by narrow strip of longer and thicker setae, Tarsus III-IV divided by wide strip of longer and wider setae. Metatarsal scopulae: I 90%; II 90%; III 65%; IV 50%. For lengths of legs and palpal segments see Table 1; legs 4, 1, 2, 3. Spination: Leg IV: metatarsus v 0-0-0 (2ap). Palp: tibia v 0-0-0 (2ap). Posterior lateral spinnerets with three segments, basal 2.07, median 0.86, digitiform apical 1.61. Lateral median spinnerets with one segment. Stridulation organ with eight primary lyra on left maxilla, eight on right; primary lyra wider from base to apex (Fig. 2). Spermatheca (Fig. 1) with two elongate asymmetrical receptacles and distant to each other, curved towards the centre and distal apex more curved and more sclerotised; apex constricted but wide. Two dorsal longitudinal folds and three ventral longitudinal folds on left receptacle, three dorsal longitudinal folds and two ventral longitudinal folds on right receptacle. Each receptacle with a single ill-defined lateral lobe on apical-inner, each lobe disposed on the most inner longitudinal fold. Colouration: carapace and legs covered with short and long bright golden setae, abdomen covered with short black setae and long reddish setae (Fig. 3).

**Table 1.** *Psalmopoeuschronoarachne* sp. nov. female holotype (ZFSQ-i11704), podomere measurements.

	Femur	Patella	Tibia	Metatarsus	Tarsus	Total
**I**	11.31	6.16	9.63	7.35	5.44	39.89
**II**	10.58	5.34	9.21	7.05	5.40	37.58
**III**	8.68	4.55	7.62	6.92	5.01	32.78
**IV**	11.03	4.85	10.03	9.74	5.03	40.68
**Palp**	7.68	4.47	5.44	–	6.44	24.03

#### Etymology.

The specific epithet is a noun in apposition referring to the combination of the Greek words *chrono* (χρόνο), in reference to time, and *arachne* (Ἀράχνη), meaning spider. The compound word refers to the adage that these spiders could “have their time counted” or reduced by impactful anthropogenic activities. The name addresses conservation concerns about the survival and prevalence of spider species in natural environments.

#### Distribution.

*Psalmopoeuschronoarachne* sp. nov. is only known from its type locality, Hacienda La Mariela at 760 m, Province of Cotopaxi, in the central area of the Cordillera Occidental of the Andes of Ecuador (Figs 11, 12).

#### Ecology.

The holotype of *Psalmopoeuschronoarachne* sp. nov. (Fig. 3) was found in the foothill evergreen forest of the Cordillera Occidental of the Andes in the Western Ecuador biogeographic province (Figs 11, 12). The spider was observed on a tree at approximately 1.5 m up from the forest floor.

### ﻿ Psalmopoeus satanas sp. nov.

Taxon classificationAnimaliaAraneaeTheraphosidae

3ACBA7E8-E8FC-57E9-9EFF-7ABC7DF4008D

https://zoobank.org/A69A2394-F71C-43E6-9414-891D4601F542

**Figure 4.**
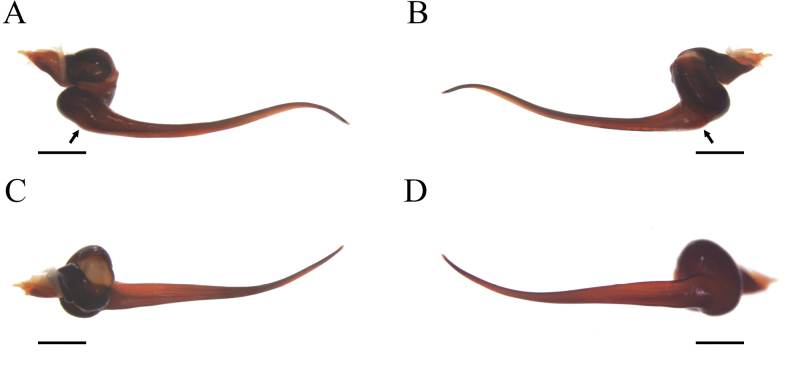
*Psalmopoeussatanas* sp. nov. male holotype (ZSFQ-i12150), palpal bulb (right hand side), arrow indicates ventral dilatation of embolus **A** prolateral view **B** retrolateral view **C** dorsal view **D** ventral view. Scale bars: 1.5 mm.

**Figure 5.**
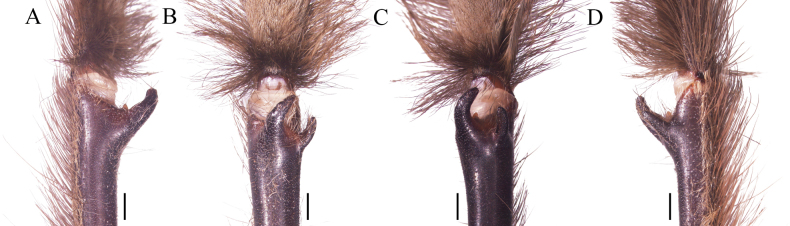
*Psalmopoeussatanas* sp. nov. male holotype (ZSFQ-i12150), tibial apophysis (left hand side) **A** retrolateral view **B** dorsal view **C** dorso-retrolateral view **D** prolateral view. Scale bars: 5 mm.

**Figure 6.**
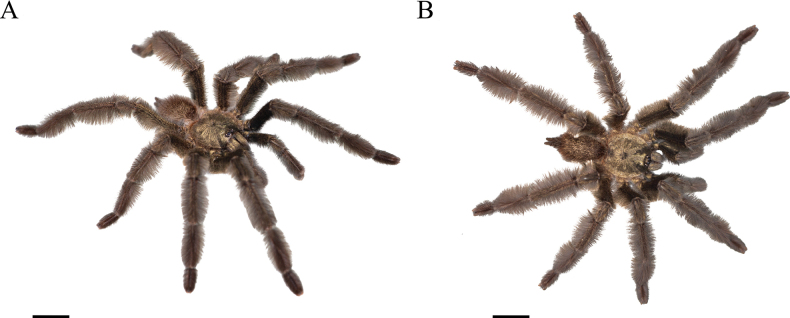
*Psalmopoeussatanas* sp. nov. male holotype (ZSFQ-i12150), live habitus **A** dorsolateral view **B** dorsal view. Scale bars: 10 mm.

**Figure 7.**
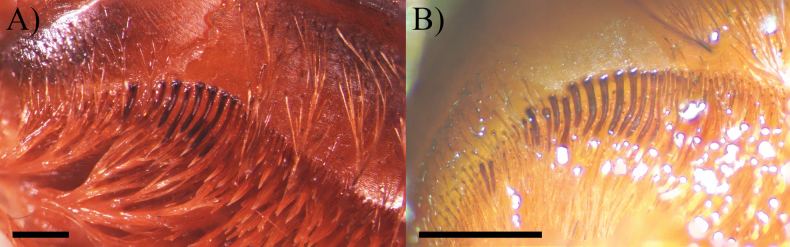
*Psalmopoeussatanas* sp. nov. Maxillae showing maxillary lyra **A** male holotype (ZSFQ-i12150) (right hand side) **B** female paratype (QCAZ-i274324) (left side). Scale bars: 0.4 mm (**A**), 0.2 mm (**B**).

**Figure 8.**
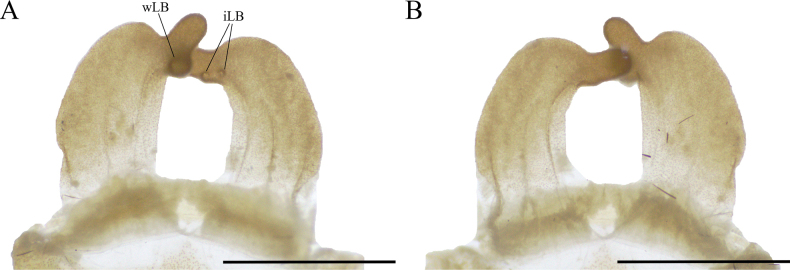
*Psalmopoeussatanas* sp. nov. female paratype (QCAZ-i274324), spermatheca **A** dorsal view **B** ventral view. Abbreviations: iLB, ill-defined lobe; wLB, well-defined lobe. Scale bars: 2 mm.

Figs 4 , 5 , 6 , 7 , 8

Psalmopoeus ecclesiasticus Pocock, 1903: Cifuentes and Bertani 2022: 217–235 (in part, misidentification).

#### Material examined.

***Holotype***: Republic of Ecuador • 1 ♂; Province of Santo Domingo de los Tsáchilas, Canton Santo Domingo, Parish of San José de Alluriquín, Reserva Otongachi - Fundación Otonga; -0.3209, -78.9513, 866 m a.s.l.; 24 May 2021; R. J. León-E, R. F. Valencia, and S. Cortese leg.; ZSFQ-i12150 (Field code: OG-Satanas).

***Paratypes***: Republic of Ecuador • 1 ♀; Province of Pichincha [= Province of Santo Domingo de los Tsáchilas], Canton Santo Domingo, Parish of San José de Alluriquín, La Magdalena; -0.2647, -79.0256, 920 m a.s.l.; 02 November 1995; B. Yangari leg.; QCAZ-i274324 (Field code: MYGA 08). Republic of Ecuador • 1 ♀; Province of Pichincha, Canton San Miguel de Los Bancos, Parish of Mindo, Los Bancos; 0.0166, -78.8833, 909 m a.s.l.; 17 December 1988; V. Navarrete leg.; QCAZ-i274323 (Field code: MYGA 40).

#### Additional material.

Republic of Ecuador • 1 sub ♀; Province of Santo Domingo de los Tsáchilas, Canton Santo Domingo, Parish of San José de Alluriquín, Reserva Otongachi - Fundación Otonga; -0.3209, -78.9517, 937 m a.s.l.; 05 October 2017; A. Tadashima leg.; ZSFQ-i12156 (Field code: AT16).

#### Diagnosis.

*Psalmopoeussatanas* sp. nov. can be distinguished from known congeners by the morphology of male palpal bulb and by female spermathecal morphology. Males of *Psalmopoeussatanas* sp. nov. can be distinguished from all other male congeners by having a slender embolus slightly curved, almost straight at distal part (Fig. 4A–D), the presence of a prominent ventral dilatation (Fig. 4A, B), and the embolus being ~ 4× the tegulum length in retrolateral view (Fig. 4B) (ventral dilatation unknown in all other congeners, for comparative measurements see Cifuentes and Bertani 2022). Additionally, males of *Psalmopoeussatanas* sp. nov. can be distinguished from *P.cambridgei*, *P.reduncus*, *P.pulcher*, *P.irminia*, *P.victori* by the absence of a distal thickening in retrolateral branch of tibial apophysis (distal thickening present in retrolateral branch of tibial apophysis in *P.cambridgei*, *P.reduncus*, *P.pulcher*, *P.irminia*, *P.victori*; see also Gabriel and Sherwood 2020: figs 13, 14; Cifuentes and Bertani 2022: figs 150–152, 187–189, 209–211, 242–244, 253–255, 280–282). Females of *Psalmopoeussatanas* sp. nov. can be distinguished from *Psalmopoeuschronoarachne* sp. nov. by having two ill-defined lobes and a single domed well-defined lobe in receptacles, apical digitiform lobe present and comparatively receptacles less curved towards the centre (Fig. 8) (only a single ill-defined lobe on each receptacle, absence of well-defined lobes, apical digitiform lobe, comparatively receptacles more curved towards the centre in *Psalmopoeuschronoarachne* sp. nov.; Fig. 1); from *P.ecclesiasticus* by having straight receptacles, distal apex curved towards the centre and overlapping, comparatively less sclerotised wider and longer apical digitiform lobe pointing upwards and only two ill-defined lateral lobe and a single domed well-defined lateral lobe on apical-inner (Fig. 8) (curved receptacles towards the centre, distal apex more curved and overlapping, receptacles comparatively with more sclerotised, thin and shorter apical digitiform lobe pointing downwards, one to four protruding well-defined lobes and a single ill-defined lobe in *P.ecclesiasticus* (Fig. 10); see also Gabriel and Sherwood 2019: fig. 1; Cifuentes and Bertani 2022: figs 224, 229); from *P.cambridgei*, *P.irminia*, *P.pulcher*, *P.langenbucheri*, *P.reduncus*, and *P.victori* by having elongated and straight receptacles with distal apex curved with the combination of only two ill-defined lateral lobe and a single domed well-defined lateral lobe on apical-inner and narrow apical digitiform lobe overlapping each other (elongated and straight receptacles with distal apex straight, comparatively more sclerotised narrow apical digitiform lobe pointing upwards but not overlapping and two to three protruding and well-defined lobes at centre in *P.cambridgei*; elongated and straight receptacles with distal apex straight, comparatively more sclerotised wider apical digitiform lobe pointing upwards but not overlapping and a single well-defined lobe at centre of each receptacle in *P.irminia*, elongated and straight receptacles with distal apex straight, comparatively more sclerotised thin apical digitiform lobe pointing upwards but not overlapping and numerous lobes at centre or lateral which reduce in size from apex to centre in *P.pulcher*; elongated and triangular receptacles with distal apex straight, comparatively more sclerotised wider and shorter apical digitiform lobe pointing upwards, not overlapping but very close and two to three well-defined lateral lobes in *P.langenbucheri*, short and triangular receptacles with distal apex straight, comparatively more sclerotised wider and shorter apical digitiform lobe pointing upwards but not overlapping and only a single ill-defined lateral lobe in *P.reduncus*; comparatively more elongated and straight receptacles with wider distal apex without receptacles in *P.victori*; see also Mendoza 2014: figs 27, 28; Cifuentes and Bertani 2022: 125, 170–175, 190, 191, 215, 245, 268–271, 283, 300, 309).

#### Description.

**Male holotype** (ZSFQ-i12150): Total length including chelicerae: 29.10. Carapace: length 12.60, width 11.62. Caput: slightly raised. Ocular tubercle: slightly raised, length 2.21, width 3.14. Eyes: ALE > PLE, PLE < AME, AME > PME, anterior eye row recurved, posterior row recurved. Clypeus: wide; clypeal fringe long. Fovea: recurved. Chelicera: length 4.25, width 2.57. Abdomen: length 11.35, width 6.32. Maxilla with 137 cuspules covering approximately 20% of the proximal edge. Labium: length 1.77, width 2.25, with 131 cuspules most separated by 1.0–2.0× the width of a single cuspule. Labio-sternal mounds joined along the entire base of the labium. Sternum: length 6.57, width 4.53, with two pairs of elongated sigilla. Tarsi I–IV fully scopulate, Metatarsal scopulae: I 95%; II 90%; III 80%; IV %, metatarsi I divided by up to half of the segment, metatarsi IV divided by a strip of longer and wider setae. For lengths of legs and palpal segments see Table 2; legs 1, 4, 2, 3. Spination: Leg II: tibia v 0-0-0 (1ap). Leg III: metatarsus v 0-0-0 (3ap). Leg IV: metatarsus v 0-0-0 (1ap). Tibia I with principal paired tibial apophysis and a short, irregular, and triangular central third apophyses, RB longer than PB, RB and PB with one megaspine (Fig. 5). Posterior lateral spinnerets with three segments, basal 3.32, median 1.14, digitiform apical 2.01. Lateral median spinnerets with one segment. Stridulation organ with 12 primary lyra on left maxilla (two of them widely separated and proximal to basal section of maxilla), ten on right (one of them slightly thinner, separated, and proximal to basal section of maxilla, slight scar on individual); other primary lyra wider from base to apex (Fig. 7). Palp (Fig. 4): tegulum length 1.53, width 0.713, embolus proximal width 0.66, length 6.41. Embolus proximal portion slightly curved with a prominent ventral dilatation in medial section. Embolus length to tegulum length: 4.18. Embolus distal third slightly curved to ventral and retrolateral sides; retrolateral curvature almost straight. Embolus tapers to the tip ending in a straight tip. Colouration: abdomen, carapace, and legs covered with short and long pale golden setae (Fig. 6). After two years in preservative, with pale grey colouration and brown setae.

**Table 2.** *Psalmopoeussatanas* sp. nov. male holotype (ZSFQ-i12150), podomere measurements.

	Femur	Patella	Tibia	Metatarsus	Tarsus	Total
**I**	16.14	6.91	14.11	13.34	6.66	56.69
**II**	14.89	6.20	13.02	12.34	5.87	52.42
**III**	12.58	5.16	10.01	11.69	6.02	44.97
**IV**	15.18	5.53	13.12	14.87	6.44	55.16
**Palp**	9.20	4.55	8.51	–	2.81	25.21

**Figure 9.**
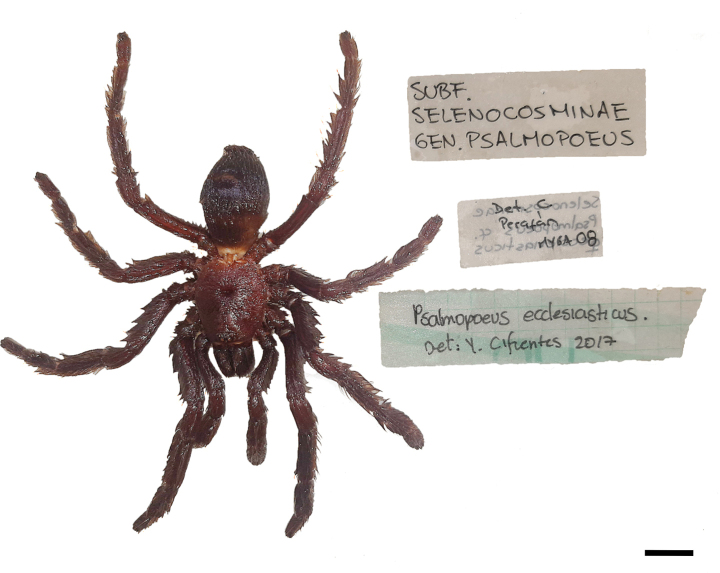
*Psalmopoeussatanas* sp. nov. female paratype, habitus and previous examinator labels. Scale bar: 15 mm.

**Female paratype** (QCAZ-i274324): Total length including chelicerae: 46.26. Carapace: length 16.45, width 15.27. Caput: slightly raised. Ocular tubercle: slightly raised, length 1.34, width 3.01. Eyes: AME > ALE, AME > PLE, PLE > PME, anterior eye row straight, posterior row slightly recurved. Clypeus: wide; clypeal fringe long. Fovea: straight. Chelicera: length 7.18, width 3.88. Abdomen: length 22.63, width 14.09. Maxilla with 170–183 cuspules covering approximately 50% of the proximal edge. Labium: length 2.46, width 2.68, with 157 cuspules most separated by 1.0–2.0× the width of a cuspule. Labio-sternal mounds joined along the entire base of the labium. Sternum: length 9.32, width 7.86, with two pairs of elongated sigilla. Tarsi I–IV fully scopulate, tarsi IV divided by wide strip of longer and thicker setae, Metatarsus IV divided by wide strip of longer and wider setae up to the half of the segment. Metatarsal scopulae: I 100%; II 100%; III 75%; IV 25%. For lengths of legs and palpal segments see Table 3; legs 4, 1, 2, 3. Spination: Leg II: tibia v 0-0-0 (1ap). Leg III: metatarsus v 0-0-0 (2ap). Leg IV: metatarsus v 0-0-0 (2ap). Palp: tibia v 0-0-0 (1ap). Posterior lateral spinnerets with three segments, basal 3.24, median 2.03, digitiform apical 2.37. Lateral median spinnerets with one segment. Stridulation organ with 15 primary lyra on left maxilla (two of them considerably thinner, widely separated, and proximal to basal section of maxilla), 13 on right (two of them considerably thinner, widely separated, and proximal to basal section of maxilla) (Fig. 7B); other primary lyra wider from base to apex. Spermatheca (Fig. 8) with two elongate asymmetrical receptacles overlapping each other, usually straight and distal apex curved towards the centre and more sclerotised; apex constricted with narrow apical lobe pointing upwards. Three dorsal longitudinal folds and ventral longitudinal folds absent on left receptacle, three dorsal longitudinal folds and ventral longitudinal folds absent on right receptacle. Left receptacle with a single well-defined lobe on apical-inner disposed on the most inner longitudinal fold. Right receptacle with two ill-defined lateral lobes on apical-inner, each lobe disposed on the most inner longitudinal fold. Colouration: after 30 years in preservative, with a dark brown colouration and pale brown setae (Fig. 9).

**Table 3.** *Psalmopoeussatanas* sp. nov. female paratype (QCAZ-i274324), podomere measurements.

	Femur	Patella	Tibia	Metatarsus	Tarsus	Total
**I**	13.68	10.01	10.22	8.05	6.73	48.69
**II**	10.49	8.00	10.53	9.03	5.06	43.11
**III**	7.72	6.26	8.14	7.22	6.28	35.62
**IV**	13.77	6.14	12.9	9.36	5.62	47.79
**Palp**	8.68	6.31	5.86	–	7.12	27.97

#### Variation.

(QCAZ-i274323) Stridulation organ with 9 primary lyra on left maxilla (two of them considerably thinner, widely separated, and proximal to basal section of maxilla), 11 on right (one of them considerably thinner, widely separated, and proximal to basal section of maxilla).

#### Etymology.

The specific epithet is a noun in apposition honouring the nickname of the holotype male *Satanas*. The members of the Mygalomorphae Research Group in the Laboratory of Terrestrial Zoology at Universidad San Francisco de Quito grew very fond of this individual during its care, in spite of the individual’s bad temperament and sporadic attacks (reason for the nickname).

#### Distribution.

*Psalmopoeussatanas* sp. nov. is known from the localities La Magdalena and Reserva Otongachi in the Province of Santo Domingo de los Tsáchilas and Los Bancos in the province of Pichincha. The new species is distributed across an altitudinal range of 866–937 m, in the north of the Cordillera Occidental of the Andes of Ecuador (Figs 11, 12).

**Figure 10.**
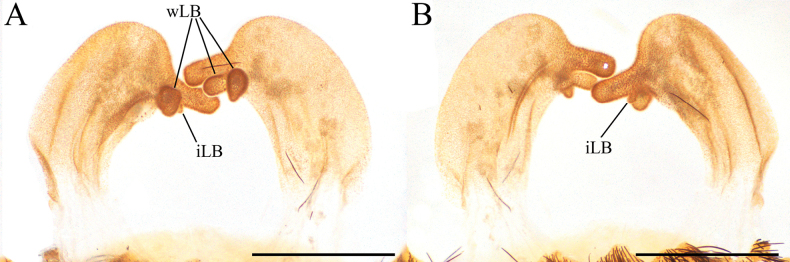
*Psalmopoeusecclesiasticus* non-type female (QCAZ-i274322), spermatheca **A** dorsal view **B** ventral view. Scale bars: 2 mm.

**Figure 11.**
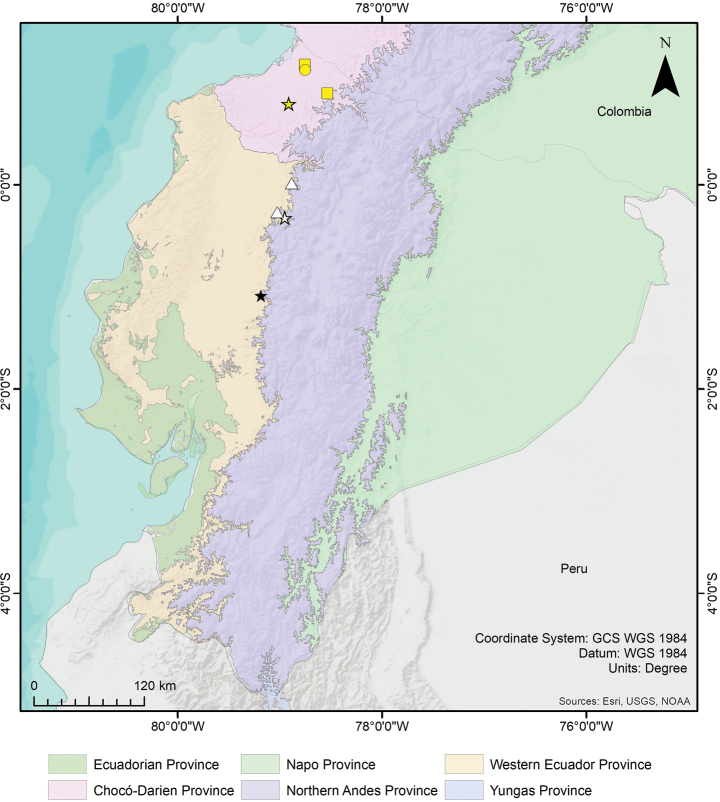
Distribution of the genus *Psalmopoeus* Pocock, 1895 in Ecuador, including biogeographical regions of Ecuador. White star = Reserva Otongachi, type locality of *P.satanas* sp. nov.; White triangle = Localities of *P.satanas* sp. nov. paratypes; Black star = Hacienda La Mariela, type locality of *P.chronoarachne*; Yellow star = Rio Sapayo, type locality of *P.ecclesiasticus*; Yellow circle = Carondelet, historical record of *P.ecclesiasticus*; Yellow squares = Additional records of *P.ecclesiasticus*.

**Figure 12.**
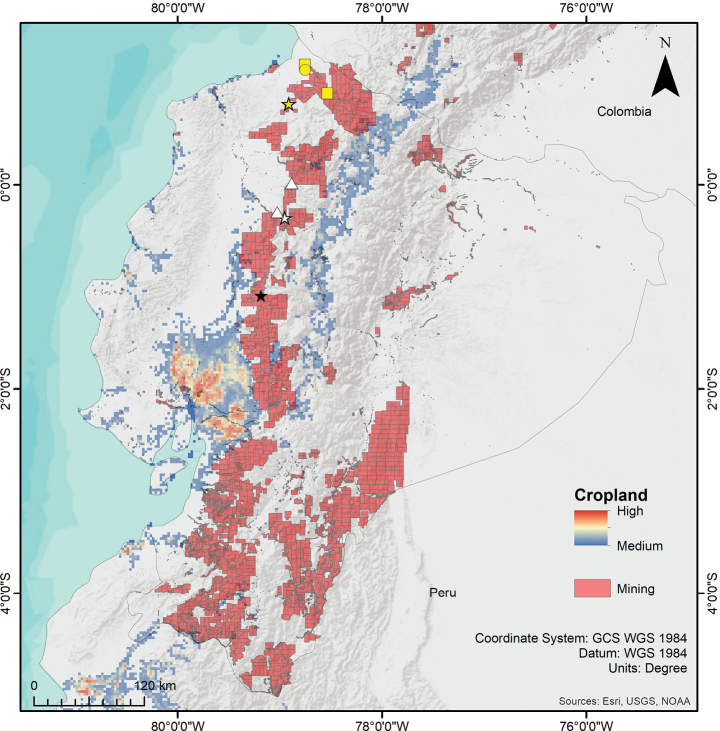
Distribution of the genus *Psalmopoeus* Pocock, 1895 in Ecuador, including mining concessions and cropland use. White star = Reserva Otongachi, type locality of *P.satanas* sp. nov.; White triangle = localities of *P.satanas* sp. nov. paratypes; black star = Hacienda La Mariela, type locality of *P.chronoarachne*; yellow star = Rio Sapayo, type locality of *P.ecclesiasticus*; yellow circle = Carondelet, historical record of *P.ecclesiasticus*; yellow squares = additional records of *P.ecclesiasticus*.

#### Ecology.

*Psalmopoeussatanas* sp. nov. is found in low montane and montane evergreen forest of the Cordillera Occidental of the Andes, in the Western Ecuador biogeographic province. The male holotype was found within a bamboo fence and exhibited defensive behaviour when observed. This behaviour then transformed into fleeing, where the spider made quick sporadic movements, nearly too fast to see.

#### Remarks.

Previously the female paratypes were examined by Carlos Perafán during his doctoral thesis about historical and actual distribution of Mygalomorphae from the northern Andes (Perafán 2017). During his revision he identified the female paratype (QCAZ-i274324) as Psalmopoeuscf.ecclesiasticus and the other female paratype (QCAZ-i274323) as *Psalmopoeus* sp., each one with a respective handwritten label (Fig. 9). Prior to this, Yeimy Cifuentes examined the same specimens for her taxonomic revision and cladistic of the subfamily Psalmopoeinae and concluded that both were *Psalmopoeusecclesiasticus*, also including a new handwritten label stating the identification of each specimen and reporting each locality for the distribution of the previously mentioned species (Cifuentes and Bertani 2022).

During the recent revision of these specimens by the first author, it was observed that the spermathecae of both specimens and also a third, also examined by Carlos Perafán and Yeimy Cifuentes which certainly is *Psalmopoeusecclesiasticus* (Fig. 10) and was collected near the type locality, were not completely cleaned and that only the left receptacle of the female paratype (QCAZ-i274323) of *Psalmopoeussatanas* sp. nov. was properly cleaned, making it impossible to observe the complete morphology of apical lobe and number of lobes. This led to both Peráfan and Cifuentes making erroneous identifications; although Carlos opted for a more conservative approach. Additionally, the right receptacle of the female paratype (QCAZ-i274323) of *Psalmopoeussatanas* sp. nov. was broken by someone who previously examined the specimen.

Morphology of tibial apophyses has been used for cladistics analysis in Psalmopoeinae and in some cases for species diagnoses (e.g., *P.langenbucheri*; Cifuentes and Bertani 2022) using some characters related to spines combination, branches development, origin of each branch, and morphology of central protuberance behind the two branches (Hüsser 2018; Cifuentes and Bertani 2022). Nevertheless, intra-specific variation has not yet been fully explored and some characters may or may not be reliable for proposing synapomorphies for previously known species or new ones; we tentatively use the distal thickening of retrolateral branch as secondary character to differentiate *P.satanas* sp. nov. from *P.cambridgei*, *P.reduncus*, *P.pulcher*, *P.irminia*, *P.victori*. Cifuentes and Bertani (2022) used the shape of the central protuberance as diagnostic character for *P.langenbucheri*. However, it should be noted that significant variation of width and length of this structure have been observed between left and right tibial apophysis in the male holotype (ZSFQ-i12150). For this reason, we encourage future researchers to evaluate intra- and inter-specific variation in order to confirm the validity of these tibial apophysis characters in species diagnosis and to evaluate morphometric aspects of other structures (e.g., leg segment ratios and spermathecae measurements; Hamilton et al. 2016; Gabriel and Sherwood 2020).

### ﻿ Psalmopoeus ecclesiasticus

Taxon classificationAnimaliaAraneaeTheraphosidae

Pocock, 1903

DFE779D5-1DCE-5256-ADD4-583FE4DD1B6E

Fig. 10

Psalmopoeus ecclesiasticus : Schmidt, Bullmer, and Thierer-Lutz (2006): 8, fig. 10.
Psalmopoeus ecclesiasticus : Gabriel and Sherwood (2019): 41, figs 1–10.
Psalmopoeus ecclesiasticus : Cifuentes and Bertani (2022): 77, figs 2, 217–235.

#### Material examined.

***Non-type material***: Republic of Ecuador • 1 ♀; Province of Esmeraldas, Canton San Lorenzo, Parish of Alto Tambo, Alto Tambo; 0.9000,-78.5333, 790 m a.s.l.; 07 December 2002; D. Salazar leg.; QCAZ-i274322 (Field code: MYGA 158).

#### Amended diagnosis.

Females of *Psalmopoeusecclesiasticus* can be distinguished from *Psalmopoeuschronoarachne* sp. nov. by comparatively having more curved receptacles towards the centre, distal apex less curved, and overlapping, receptacles with apical digitiform lobe overlapping, one to four protruding well-defined lobes, and a single ill-defined lobe (Fig. 10) (comparatively less curved receptacles towards the centre and distant to each other, distal apex more curved and not overlapping, receptacles with only a single ill-defined lateral lobe on apical-inner and apical digitiform lobe absent in *Psalmopoeuschronoarachne* sp. nov.; Fig. 1); from *Psalmopoeussatanas* sp. nov. by having curved receptacles towards the centre, distal apex more curved and overlapping, receptacles comparatively with more sclerotised, thin and shorter apical digitiform lobe pointing downwards, one to four protruding well-defined lobes and a single ill-defined lobe (straight receptacles, distal apex curved towards the centre and overlapping, comparatively less sclerotised wider and longer apical digitiform lobe pointing upwards and only two ill-defined lateral lobe and a single domed well-defined lateral lobe on apical-inner in *Psalmopoeussatanas* sp. nov.; Fig. 8); from *P.cambridgei*, *P.irminia*, *P.pulcher*, *P.langenbucheri*, *P.reduncus*, and *P.victori* by having elongated curved receptacles towards the centre, distal apex curved and overlapping, thin and shorter apical digitiform lobe pointing downwards, one to four protruding well-defined lobes, and a single ill-defined lobe disposed on the central longitudinal fold (elongated and straight receptacles with distal apex straight, comparatively more sclerotised, narrow, and elongated apical digitiform lobe pointing upwards and not overlapping and two to three protruding and well-defined lobes at central longitudinal fold in *P.cambridgei*; elongated and straight receptacles with distal apex straight, comparatively more sclerotised wider and shorter apical digitiform lobe pointing upwards but not overlapping and a single domed well-defined lobe at centre of each receptacle in *P.irminia*, elongated and straight receptacles with distal apex straight, comparatively more sclerotised thin apical digitiform lobe pointing upwards but not overlapping and numerous lobes at centre or lateral which reduce in size from apex to centre in *P.pulcher*; elongated and triangular receptacles with distal apex straight, comparatively more shorter apical digitiform lobe pointing upwards, not overlapping but very close to each other and two to three well-defined lateral lobes in *P.langenbucheri*, short and triangular receptacles with distal apex straight, comparatively more sclerotised wider and shorter apical digitiform lobe pointing upwards but not overlapping and only a single ill-defined lateral lobe in *P.reduncus*; comparatively more elongated and straight receptacles with wider distal apex without receptacles in *P.victori*; see figures in Mendoza 2014: figs 27, 28; Cifuentes and Bertani 2022: 125, 170–175, 190–191, 215, 245, 268–271, 283, 300, 309)

#### Remarks.

During a recent visit to the QCAZ collection a female of *P.ecclesiasticus* was examined by PPR. The specimen was collected in the locality Alto Tambo, almost *ca.* 43 km SW from the type locality of this species. Herein we illustrate the spermatheca of this specimen, demonstrating intraspecific variation, not able to be shown in previous works (Gabriel and Sherwood 2019; Cifuentes and Bertani 2022); accordingly, we provide an updated diagnosis for the females of *P.ecclesiasticus*.

#### Conservation.

Little is known about the actual population density of *P.chronoarachne* sp. nov., and *P.satanas* sp. nov. Through a comparison of the known distribution of each species and the most probable overlapping anthropogenic threats, we found that mining concessions occupy the entire distribution of the species evaluated and expand further, demonstrating a severe distributional threat (Fig. 12). This, considering that these areas close to the known distribution of each species, may represent potential distributions of the populations. Likewise, another threat that was considered was agriculture and croplands. Even though it does not seem to overlap with the distribution of the species, this plausible threat is very close to the known localities. Additionally, we must remark that the data used for cropland distribution in Ecuador is not current and for many localities that occupy these species, cropland and livestock grasslands are present in larger than currently given projections (pers. obs.). In fact, the type localities of *Psalmopoeuschronoarachne* sp. nov. and *P.satanas* sp. nov. are both surrounded by cropland and livestock grassland (Brito-Zapata pers. comm. 09 May 2023; RJL-E pers. obs.) but this is not officially registered.

According to the IUCN (2001) criteria for the Red List Categories, in poorly known taxa and where the population statuses of species are not known in detail (see Discussion), background information on habitat deterioration and other causal factors can be used for assigning any threat category. Based on the information previously mentioned, herein we propose that *Psalmopoeuschronoarachne* sp. nov. should be placed in the *Critically Endangered* category based on the criteria combination B2abiii by taking in reference that the estimated area of occupancy estimated for this species is less than 10 km^2^, demonstrating severe fragmentation caused by cropland and mining concessions. Likewise, the territory is inferred to be on a steady decline in area of occupancy and quality of habitat. Similarly, *Psalmopoeussatanas* sp. nov. could also be classified in the *Critically Endangered* category based on the criteria combination B1abiii by taking in reference that the area of occupancy estimated for this species is ~ 83 km^2^, severely fragmented by cropland and mining concessions and following an inferred continuing decline in the available area of occupancy and quality of habitat.

## ﻿Discussion

*Psalmopoeuschronoarachne* sp. nov. appears to be endemic to its type locality in Hacienda La Mariela in the Pangua canton. Pangua is located slightly west between the Quilotoa and Chimborazo massifs geographically (Stern 2004), a region recognised for its high biodiversity, critical to numerous threatened species of amphibians, birds, mammals, among others, that rely on its ecosystems. The locality is ecologically similar to the Reserva Ecológica Ilinizas, especially its subtropical forests. However, there exists a difference in beta biodiversity because the locality is in an intermediate zone between the subtropical forests of Reserva Ecológica Ilinizas and the arid environments of Reserva de Producción de Fauna Chimborazo (Tapia Armijos 2016), relatively low in elevation (760 m), but intercepting two diametrically distinct ecological regions.

Nonetheless, although the region is relatively ecologically unique, because Pangua is not within the bounds of any governmental ecological reserve, it is highly threatened by both legal (Fig. 12) and illegal mining operations that extract metals such as copper, silver, and gold, introducing pollutants to its ecosystems (Cooper and Jolly 1970; Ngole-Jeme and Fantke 2017; Liu et al. 2020). Habitat fragmentation due to the expansion of urban and agricultural zones, accompanied by the concurrent introduction of non-native species, represents other threats the region’s ecosystems face (Pimm 1987; Simberloff et al. 2005; Ricciardi and Cohen 2007). Thus, given these escalating threats to the ecosystems of Pangua, it is essential to consider that this species meets the aforementioned conservation categories and should be classified as C*ritically Endangered* within the criteria combination B2abiii.

Similarly, in the case of *P.satanas* sp. nov., the species also appears to be endemic to the western foothill forest near San José de Alluriquin and Mindo. This geographic region is affected by the Toachi and Pilatón rivers which flow from the Corazón volcano and affect the terrain to form orographic formations such as Macuhi, Pasayambo, Yunguilla, and Zarapullo (Arcos Argoti 2011). Because of this topography, the region is irregular, complex, and prone to high endemism; thus, especially threatened (Sonne et al. 2022).

As aforementioned for *P.chronoarachne* sp. nov., this region is also unprotected by governmental ecological reserves. However, various private and communal protected areas, like La Hesperia, Fundación Otonga, and Yunguilla nearby, may serve as sanctuaries. This region faces habitat loss due to fragmentation, deforestation, and both legal (Fig. 12) and illegal mining (La Florida Mining Concession). Consequently, the introduction of pollutants, non-native species, or habitat fragmentation is plausible and a latent threat (Cooper and Jolly 1970; Pimm 1987; Simberloff et al. 2005; Ricciardi and Cohen 2007; Ngole-Jeme and Fantke 2017; Liu et al. 2020). As a consequence, similar to the case of *P.chronoarachne* sp. nov., it is essential to consider that *P.satanas* sp. nov. could also be classified as *Critically Endangered*, within the criteria combination B1abiii.

Consequently, it is essential to consider the potential loss of both *P.chronoarachne* sp. nov. and *P.satanas* sp. nov. and the ecological consequences that would result from their extinctions. These species are the only arboreal clades of theraphosid spiders in the region and thus may serve essential roles in the stratified micro-ecosystems in their respective areas.

To avoid this loss in Ecuadorian biodiversity, it is essential that these species be considered legally and that stricter regulations and penalties for illegal mining or other extracting-related activities, including specimen smuggling, be implemented to discourage such practices. Likewise, the engaging and educating of local communities about the importance of biodiversity conservation is essential to avoid further extinction and to educate about the potential economic benefits derived from ecotourism initiatives. Finally, it is important to consider that the areas in which these arthropods live are not under legal protection. The implementation of protected areas in these localities is essential to maintain the remaining population of these endangered species, and to encourage research on the remaining undescribed or unknown tarantula species in the area.

As a final point, we would like also to emphasise the latent threat of the illegal pet trade of wild tarantulas as a reason for wild population declines of tarantulas (Fukushima et al. 2019). This issue has been present in tarantula field collection since the peak of the tarantula pet hobby trade in the 1980s in North America and Europe (Smith 2020). Considering the publications of some hobby taxonomists who described novel species or made taxonomic treatments of Ecuadorian species based on pet-trade specimens (obviously wild caught), it is inferred that the issue has been ongoing for more than 32 years in Ecuador (Schmidt 1986, 1993, 1995a, 1995b, 2002, 2003a, 2003b; Kirk 1990; Schmidt and Bauer 1996; Bauer and Antonelli 1997; Peters 2003, 2005; Bullmer et al. 2006; Schmidt et al. 2006). Although this series of publications encouraged research on Ecuadorian tarantulas previously ignored for centuries, they also functioned as catalysts within the exotic pet-trade hobby, aiding in obtaining these species and further encouraging people to collect undescribed species. During this time, the sale, purchase, and study of these specimens in other countries was not considered illegal nor was it regulated by international institutions or hobbyist societies. In Ecuador since 1981 the fauna and flora become part of the domain of the state by the addition of Art. 74 in which implied penal actions against those who extract, commercialise, transport, and acquire wildlife and derived products (Corte Suprema de Justicia 1981), meaning that the first steps of Ecuadorian specimens present in the tarantula pet market started from illegal extractions.

“Official” evidence of illegally trafficked Ecuadorian tarantulas reported by Ecuadorian institutional authorities is limited: only two reports highlighting the confiscation of unknown species of tarantulas in Ecuador were made public in 2018 and 2021 by Ministerio del Ambiente, Agua y Transicion Ecológica and local newspapers (PP-R pers. obs.). However, it is relatively easy to find Ecuadorian specimens belonging to the genera *Amazonius* Cifuentes & Bertani, 2022, *Avicularia* Lamarck, 1818, *Megaphobema* Pocock, 1901, *Cyclosternum* Ausserer, 1871, *Cymbiapophysa* Gabriel & Sherwood, 2020, *Tapinauchenius* Ausserer, 1871, *Thrixopelma* Schmidt, 1994, *Neischnocolus* Petrunkevitch, 1925, *Pamphobeteus* Pocock, 1901, and *Psalmopoeus* Pocock, 1895 being available for sale in various websites and Facebook group*s* (PP-R pers. obs.). It is important to understand that although some of these specimens have been bred in captivity, wild specimens are still being commercialised. It is clear that the knowledge of the ecologies and trophic dynamics of tarantulas in Ecuador, and the world, can still be improved upon. However, it is likely that when a thorough evaluation of the conservation status of each known species will be achieved, many of these will meet the critical categories within the IUCN criteria. We encourage future work by Ecuadorian and international researchers, organisations, and governments to effectively understand the reality about the threat of tarantula smuggling and the required conservation status of each species in the country.

## Supplementary Material

XML Treatment for Psalmopoeus chronoarachne

XML Treatment for Psalmopoeus satanas

XML Treatment for Psalmopoeus ecclesiasticus

## ﻿Additional information

### Author ORCIDs

Pedro Peñaherrera-R. https://orcid.org/0000-0001-9285-3403

Roberto J. León-E. https://orcid.org/0000-0002-9710-234X

### Data availability

All of the data that support the findings of this study are available in the main text.
